# Calpains in cyanobacteria and the origin of calpains

**DOI:** 10.1038/s41598-022-18228-2

**Published:** 2022-08-16

**Authors:** Dominika Vešelényiová, Lenka Hutárová, Alexandra Lukáčová, Mária Schneiderová, Matej Vesteg, Juraj Krajčovič

**Affiliations:** 1 Department of Biology, Faculty of Natural Sciences, University of Ss. Cyril and Methodius in Trnava, Námestie J. Herdu 577/2, 917 01 Trnava, Slovakia; 2 Department of Biology and Ecology, Faculty of Natural Sciences, Matej Bel University, 974 01 Banská Bystrica, Slovakia

**Keywords:** Computational biology and bioinformatics, Evolution, Microbiology

## Abstract

Calpains are cysteine proteases involved in many cellular processes. They are an ancient and large superfamily of enzymes responsible for the cleavage and irreversible modification of a large variety of substrates. They have been intensively studied in humans and other mammals, but information about calpains in bacteria is scarce. Calpains have not been found among Archaea to date. In this study, we have investigated the presence of calpains in selected cyanobacterial species using in silico analyses. We show that calpains defined by possessing CysPC core domain are present in cyanobacterial genera *Anabaena*, *Aphanizomenon*, *Calothrix*, *Chamaesiphon*, *Fischerella*, *Microcystis*, *Scytonema* and *Trichormus*. Based on in silico protein interaction analysis, we have predicted putative interaction partners for identified cyanobacterial calpains. The phylogenetic analysis including cyanobacterial, other bacterial and eukaryotic calpains divided bacterial and eukaryotic calpains into two separate monophyletic clusters. We propose two possible evolutionary scenarios to explain this tree topology: (1) the eukaryotic ancestor or an archaeal ancestor of eukaryotes obtained calpain gene from an unknown bacterial donor, or alternatively (2) calpain gene had been already present in the last common universal ancestor and subsequently lost by the ancestor of Archaea, but retained by the ancestor of Bacteria and by the ancestor of Eukarya. Both scenarios would require multiple independent losses of calpain genes in various bacteria and eukaryotes.

## Introduction

Calpains (EC 3.4.22.17) are an ancient superfamily of cytosolic, non-lysosomal cysteine proteases. Proteases are often synthesised as inactive precursors possessing N-terminal inhibitory propeptides which prevent uncontrolled preoteolysis, and they are activated by cleavage of the propeptide. Other functions of these propeptides include functional modulation, liganding, correct protease folding and compartmentalization^1^. Such N-terminal propeptides are absent from calpains and instead, they are activated by binding of Ca^2+^ ions like many other cysteine proteases^2^.

**Figure 1:**
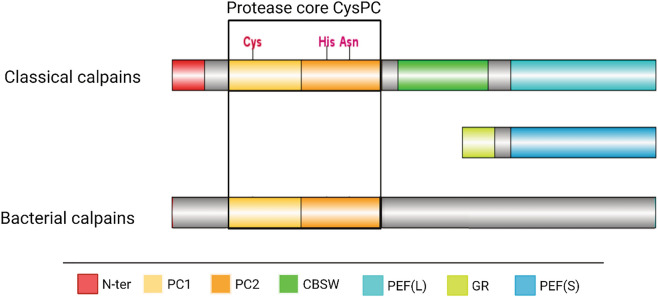
Structural comparison of classical and non-classical calpains. Classical eukaryotic calpains are heterodimers composed of large and small subunits. Each subunit is composed of conserved domains. Large subunit of classical calpains is composed of N-terminal domain, CysPC domain composed of protease core domains 1 and 2 (PC1 and PC2), calpain-type β-sandwich (CBSW) domain and a penta-EF hand domain of the large subunit, PEF(L). Small subunit contains two conserved domains: penta-EF hand domain PEF(S) and a glycine-rich region (GR). Classical calpains are absent from bacteria. Non-classical calpains present in some bacteria (but also in some eukaryotes) are monomers, typically with only a single conserved domain—calpain catalytic domain CysPc composed of PC1 and PC2.

The first calpain was discovered by Guroff (1964) when he purified a calcium-activated enzyme present in a soluble fraction of the rat brain^3^. Other calpains have been later identified in various organisms including mammals, invertebrates, plants and fungi^4^ as well as in some bacteria, but they have not been found in Archaea^5^. Calpains are divided into two groups based on their structure: classical and non-classical ones (Fig. 1). Classical eukaryotic calpains are composed of large and small subunits that are assembled to a functional heterodimer after activation by calcium ions. The large subunit of classical calpains is composed of N-terminal domain, catalytic CysPC domain composed of protease core domains 1 and 2 (PC1 and PC2), C2-like domain and a penta-EF hand domain of the large subunit, PEF(L). Small subunit contains only two conserved domains: penta-EF hand domain of the small subunit, PEF(S), and a glycine-rich region (Fig. 1). Calpains found in bacteria (but also some eukaryotic ones) belong to the group of non-classical calpains, they are monomers lacking the small subunit, and they have in common with other calpains only the catalytic calpain domain CysPC^5^. The PEF domains are specialised for calcium binding, but the CysPC domain can bind Ca^2+^ as well^6^.

Although the number of calpain studies has dramatically increased in recent years, they have focused mainly on mammalian classical calpains, because of their biomedical and clinical importance^7^. Calpains are involved in the development of various diseases including limb-girdle muscular dystrophy^8,9^, type II diabetes^10^, neurodegenerative disorders^11,12^ and cancer^13^. The information about calpains in bacteria and unicellular eukaryotes remains scarce, with the exception of calpains found in yeasts which are relatively well-characterised^14,15^.

Cyanobacteria are one of the most ancient and major groups of Gram-negative bacteria. They obtain energy by photosynthesis. Cyanobacteria are the only diazotrophs producing oxygen as a by-product of the photosynthetic process^16^. They are an enormously diverse group with high adaptive capacity and many species have the ability to tolerate extreme conditions^17^. Therefore, they have colonised almost all habitats on the Earth with the access to sunlight and they play a significant role in biochemical processes in nature^18^. The chloroplasts of eukaryotic supergroup Archaeplastida comprising glaucophytes, and red and green algae including land plants^19^ have originated from cyanobacteria in the process termed primary endosymbiosis^20–22^.

Due to the advances in genomics, transcriptomics and proteomics, the genetic makeup of cyanobacteria has been studied more intensively and their significance in biotechnological applications has increased. Cyanobacteria can be a source of bioactive compounds including pharmaceuticals and toxins^23^. Several calcium binding proteins (CaBPs) have been discovered in cyanobacteria. These proteins play a significant role in bacterial cells, mainly in processes such as cell division and development, motility, homeostasis, stress response, secretion, molecular transport, cellular signalling and host–pathogen interactions^24^. Nevertheless, the information about cysteine proteases from the calpain superfamily in cyanobacteria remains limited.

In this study, we have conducted bioinformatic search for calpain homologs in proteomes of various selected cyanobacterial species, mainly colonising extreme environments and species with biotechnological significance. The putative interacting partners of cyanobacterial calpains have also been identified in silico. We have also performed the phylogenetic analysis of calpain core CysPC domain to infer the phylogenetic position of the identified cyanobacterial calpains.

## Results

Since information about calpains in bacteria is still limited, we decided to search for these cysteine proteases in 50 selected cyanobacterial species (Supplementary Table S1). Using HMM of calpain catalytic domain (CysPC) and subsequent conserved domain prediction by CDD and Pfam, we identified 13 putative calpain sequences (Table 1) in 10 of 50 cyanobacterial species (10/50; 20%), seven species (*Anabaena minutissima, Aphanizomenon flosaquae, Calothrix parasitica, Fischerella thermalis*, *Fischerella muscicola*, *Scytonema hofmannii* and *Trichormus variabilis*) belonging to order Nostocales, one species (*Microcystis aeruginosa*) belonging to Chroococcales and two species (*Chamaesiphon minutus* and C*hamaesiphon polymorphus*) to Synechococcales. Three putative calpains were identified in *S. hofmanii*, two in *F. thermalis*, while only one calpain was identified in other cyanobacterial species.

**Table 1:**
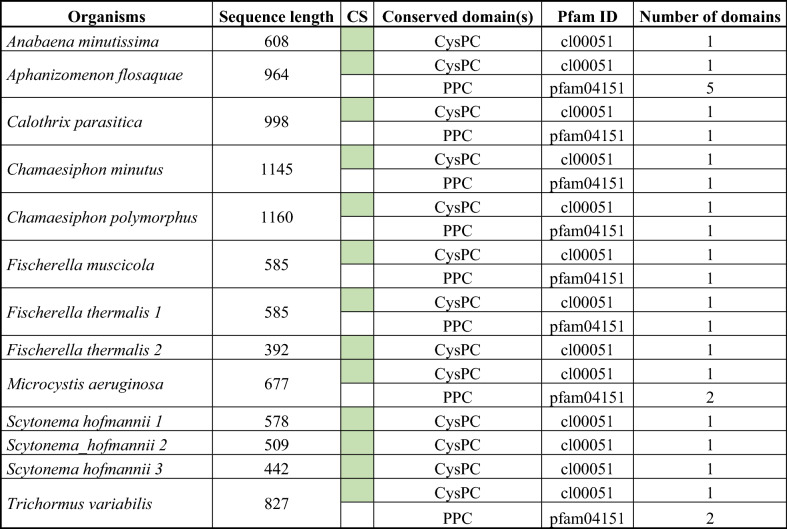
Information about calpains found in cyanobacteria.

All sequences possess a single catalytic core domain of calpains (CysPC). Most sequences also possess one, two or five bacterial pre-peptidase C-terminal domains (PPC). All identified CysPC domains contain three catalytic sites (CS) typical for calpains.

**Figure 2:**
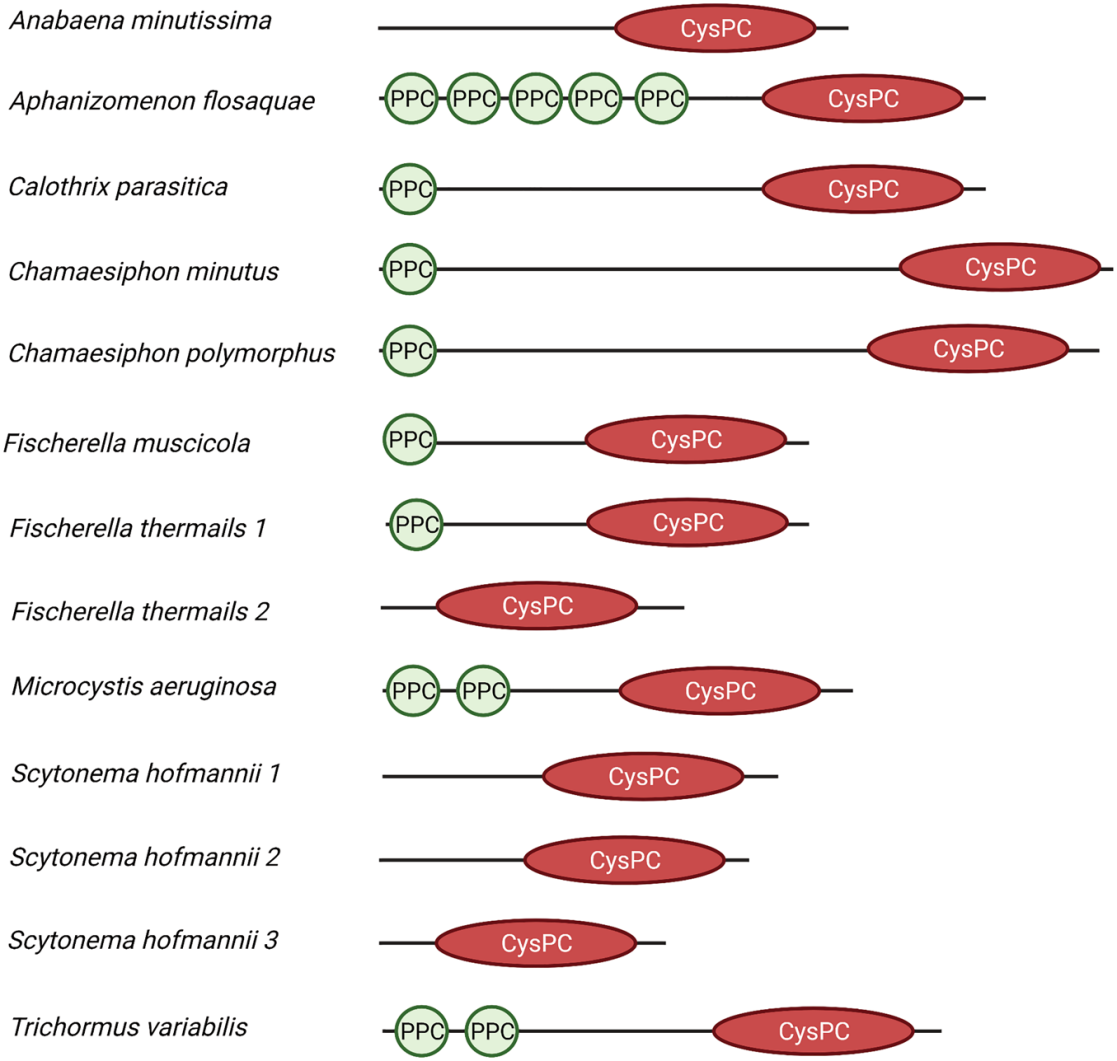
Domain structure of the identified cyanobacterial calpains. All calpains contain CysPc conserved domain (red ellipse) with a catalytic triad C, H, N typical for calpains. Some calpains possess the second conserved domain, PPC (green circle), which can be present in several copies.

The Supplementary Table S2 shows the sequences of 13 cyanobacterial calpains identified in this study. Their sequence length ranges from 382 amino acid residues in *F. thermalis* to significantly longer sequences in *C. minutus* and *C. polymorphus* (1145 and 1160 amino acid residues, respectively). The domain structures of all 13 putative calpains analysed using CDD and Pfam are summarised in Table 1. All identified calpains contain conserved CysPC domain at the C-terminus and the most of them also contain single or multiple bacterial pre-peptidase C-terminal domains (PPCs) at the N-terminus (Table 1, Fig. 2).

**Figure 3:**
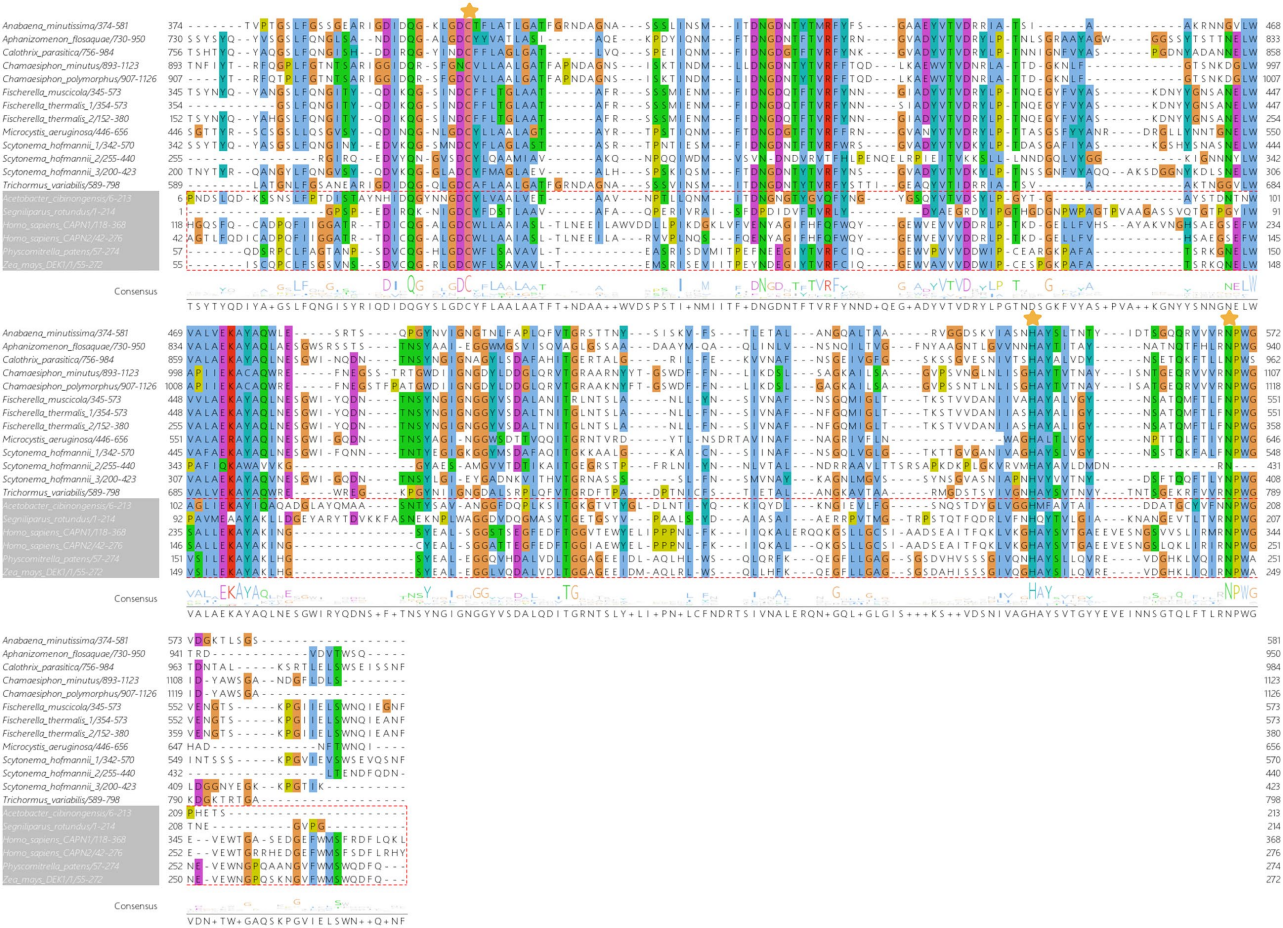
Multiple sequence alignment of CysPC domains present in the identified cyanobacterial calpains and the generated sequence logo. Two another bacterial CysPCs, CysPCs present in human calpains 1 and 2, and in DEK1 calpains from *Zea mays* and *Physcomitrella patens* were also included in the alignment. The presence of conserved C, H and N residues characteristic for CysPC domains is marked by yellow asterisks.

**Figure 4:**
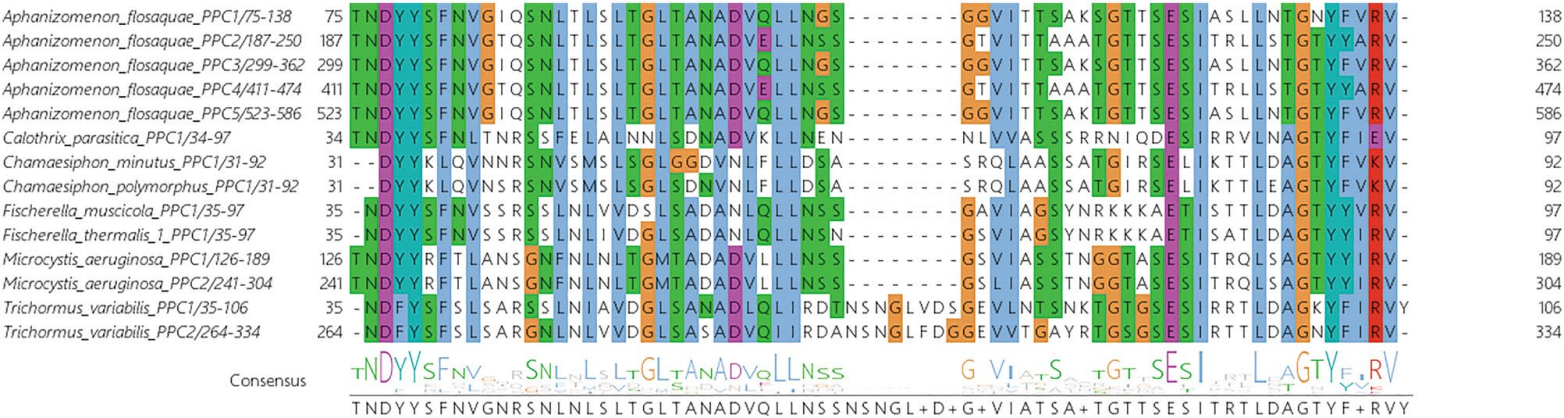
Multiple sequence alignment of PPC domains present in cyanobacterial calpains and the generated sequence logo.

The alignment of CysPC domains from 13 cyanobacterial calpains, two another bacterial calpains, human calpains CAPN1 and CAPN2, and from two plants DEK1 calpains, and the sequence logo generated from this alignment is presented in Fig. 3. By definition, all CysPC domains should share a catalytic triad of amino acids typical for calpains—Cys (C), His (H) and Asn (N). All these three residues were correctly aligned for all 13 cyanobacterial CysPC domains (Fig. 3). Figure 4 shows the alignment of PPC domains present in cyanobacterial calpains.

Although most calpains are cytosolic, a few of eukaryotic calpains can be also found in organelles such as mitochondria, e.g. human calpain 10^4,25^ or they are transmembrane proteins in plasma membrane as in the case of plant calpain DEK1^26,27^. Thus, we also performed prediction of transmembrane regions in cyanobacterial calpains. TMHMM did not identify transmembrane regions in any of cyanobacterial calpains suggesting their cytosolic localization.

Smart BLAST was used to evaluate whether the identified sequences are considered to belong to the calpain superfamily. All 13 sequences show reasonable similarity with members of this superfamily. However, the level of sequence identity is relatively low (~ 30%). This might be due to the lack of annotated bacterial calpain sequences in public databases and only a limited number of well-studied calpains from unicellular eukaryotes and bacteria.

**Figure 5:**
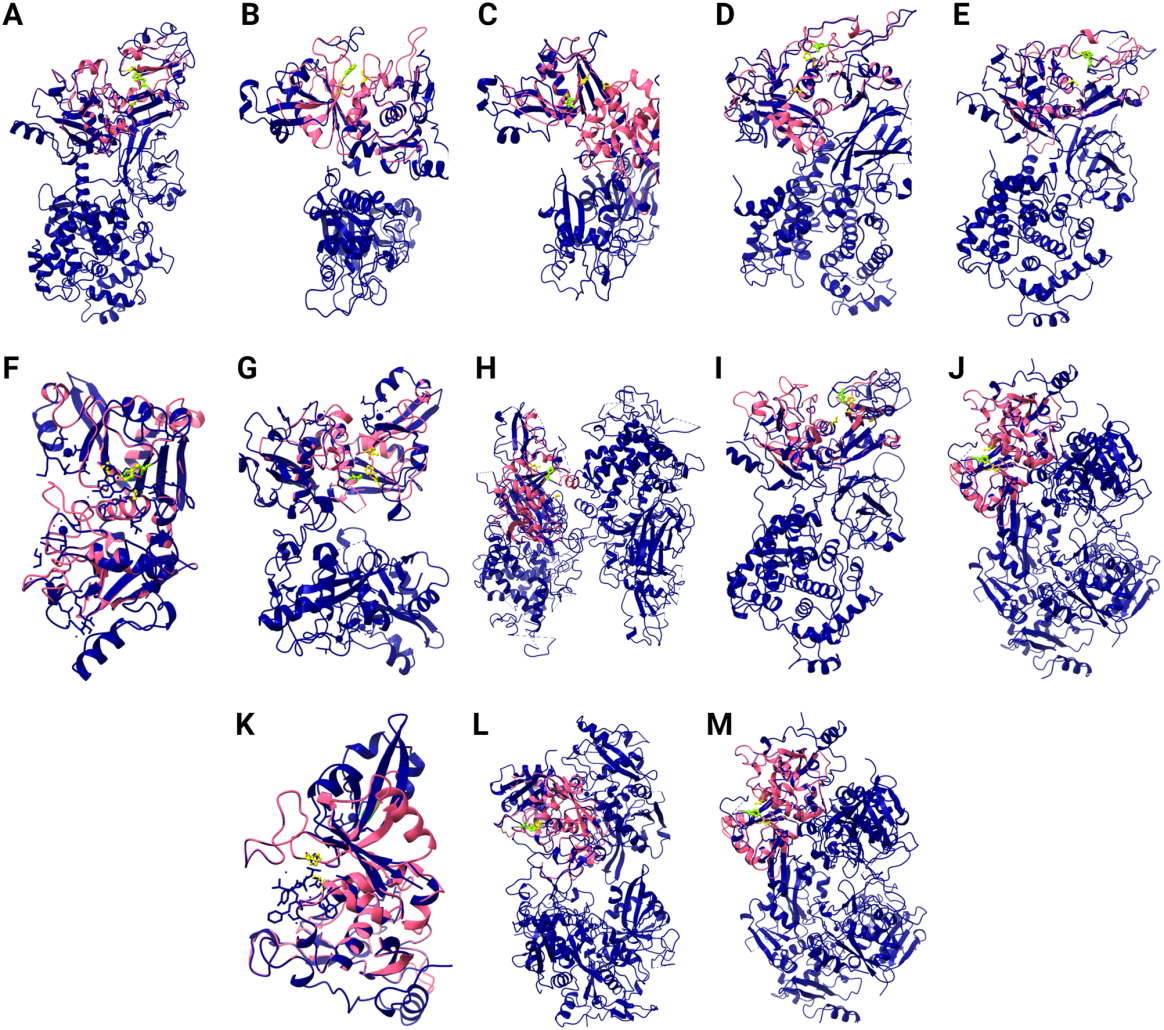
3D structure modelling of cyanobacterial CysPC domains. The comparison of the template structure obtained from Protein Data Bank (dark blue) and the predicted 3D structure of the cyanobacterial CysPC domain (pink). Catalytic amino acid triad C, H, N is shown in yellow. (**A**) *Anabaena minutissima*, (**B**) *Aphanizomenon flosaquae*, (**C**) *Calothrix parasitica*, (**D**) *Chamaesiphon minutus*, (**E**) *Chamaesiphon polymorphus*, (**F**) *Fischerella muscicola*, (**G**)* Fischerella thermalis* 1, (**H**) *Fischerella thermalis* 2, (**I**) *Microcystis aeruginosa*, (**J**) *Scytonema hofmannii* 1, (**K**) *Scytonema hofmannii* 2, (**L**) *Scytonema hofmannii* 3, M) *Trichormus variabilis*.

We performed also homological modelling of the 3D structure of each identified cyanobacterial CysPC domain. The results are summarised in Supplementary Table S3. The modelled structure was aligned with the appropriate Protein Data Bank (PDB) template, and the alignment was evaluated based on the number of aligned amino acid residues and Root mean square deviation (RMSD). All 3D structures show significant similarity with the template with RMSD > 1 for all modelled CysPCs (Supplementary Table S4). 3D structures are shown in Fig. 5.

Using String DB^28^, we predicted the putative interactions of cyanobacterial calpains with other proteins. Almost 40% of interaction partners of cyanobacterial calpains predicted by String DB were putative cyanobacterial proteins currently missing annotation in public databases. Putative interaction partners of cyanobacterial calpains are shown in Supplementary Fig. S1.

**Figure 6:**
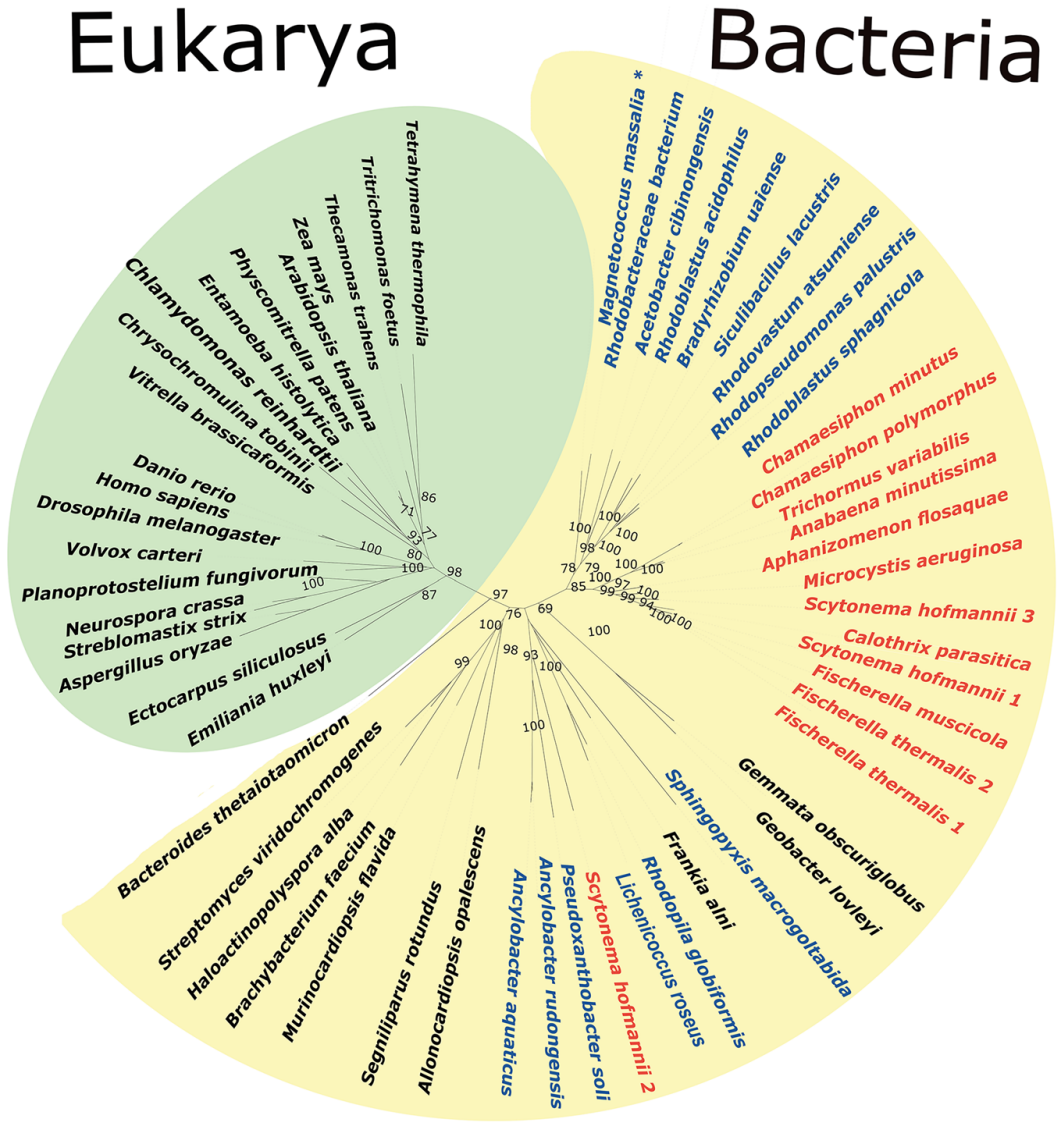
Unrooted phylogenetic tree of calpain catalytic CysPC domains. Cyanobateria are in red and alphaproteobacteria in blue. *Recent phylogenetic studies of alphaproteobacteria suggested that magnetotactic bacteria including *Magnetococcus* spp. should be excluded from alphaproteobacteria and placed into the separate class Magnetococcia^56^.

To determine evolutionary relationships between cyanobacterial calpains and calpains present in other bacteria and eukaryotes, we performed phylogenetic analysis of the CysPC domain. In contrast to other parts of calpain sequences, CysPC domain is highly conserved in all calpains. It consists of approximately 350 amino acid residues. The results of phylogenetic analysis are shown in Fig. 6. Bacterial and eukaryotic CysPC domains are clearly separated into two monophyletic clusters. All cyanobacterial calpains, except for *S. hofmanii 2*, form a monophyletic cluster within bacteria.

## Discussion

We have searched for the presence of calpains in proteomes of 50 cyanobacterial species and we have identified calpains in 10 of them based on HMM of the catalytic CysPC domain typical for calpains proteins. The number of identified cyanobacterial species possessing calpains is relatively low, but as it has been shown previously, cyanobacteria are a highly diverse group and their genome content varies significantly even at the species and strain levels^29^. CysPC domain is in cyanobacteria often associated with PPC domain (Table 1, Fig. 2), which is typically present in bacterial secreted proteins at their C-terminus^30^, while in cyanobacterial calpains, it is found at the N-terminus. The transmembrane helical regions are absent from all putative cyanobacterial calpains suggesting their cytosolic localisation. These findings are consistent with the study of calpains in other bacteria that also possess PPC at the N-terminus and do not possess any predictable transmembrane regions^5^.

Calpains are known to be involved in many cellular processes in multicellular eukaryotes such as aleurone bilayer development and positional cell division in plants^31^, and brain function, memory formation and the development of many pathological processes in mammals^7^. Calpains cleave a wide range of substrates, among which are e.g. protein kinases, receptor molecules and proteins involved in signal transduction. It has been proposed that calpains play main role in regulation of cell signalling rather than in protein digestion^32,33^. However, their function in bacteria remains unknown.

The predicted interaction partners of identified cyanobacterial calpains differ significantly among studied cyanobacterial species. None of them has been predicted to interact with calpains in all cyanobacterial species and only few of them have been commonly predicted for two, three or four species. Methionine synthase is putatively interacting with calpains in four cyanobacterial species, while S8 peptidase and glycoside hydrolase family 3 proteins (such as beta-N-acetylhexosaminidase) with calpains in three cyanobacterial species. SecA involved in protein translocation across cytoplasmic and thylakoid membrane, TamB (a component of the translocation and assembly module autotransporter complex) and collagen triple helix repeat protein have been identified as putative calpain interacting partners only in two cyanobacterial species. Other annotated proteins putatively interacting with cyanobacterial calpains have been predicted only for a single cyanobacterial species and almost 40% of predicted interacting partners have been non-annotated proteins (Supplementary Fig. S1). Based on these results, it is currently difficult to draw any meaningful conclusion about a function of cyanobacterial calpains. The predicted interaction partners and the function of cyanobacterial calpains can be experimentally verified in the future.

We also conducted phylogenetic analysis of calpain core CysPC domain to infer the phylogenetic position of cyanobacterial calpains. The phylogenetic analysis revealed the monophyly of bacterial as well as of eukaryotic CysPCs with bootstrap support 97 and 98, respectively (Fig. 6). No horizontal gene transfers of CysPC domain from bacteria to eukaryotes or vice versa were detected using our taxon sampling. This is consistent with the results of Rawlings (2015) whose phylogenetic analysis identified only two recent horizontal gene transfers from eukaryotes to bacteria and no recent horizontal gene transfer from bacteria to eukaryotes^5^. The branching order within the domain Bacteria and within the domain Eukarya does not correspond to real evolutionary relationships of bacterial and eukaryotic taxonomic groups, respectively. CysPC is thus unlikely to be a suitable marker for inferring the evolutionary relationships between organisms and it is also possible that several horizontal transfers of calpains have occurred within bacteria as well as within eukaryotes.

With the exception of *S. hofmannii 2*, all cyanobacterial CysPC domains are a monophyletic group within bacterial CysPC domains (Fig. 6). The alignment of cyanobacterial CysPC domains also confirms that CysPC domain 2 from *S. hofmannii* is the most divergent in comparison to other cyanobacterial CysPC domains (Fig. 3). The tree topology also disproves the hypothesis that cyanobacteria, from which chloroplasts of Archaeplastida evolved, were the endosymbiotic donors of archaeplastidial calpains.

The explanation of the origin of eukaryotic calpains depends on the opinion about the origin of eukaryotes themselves. The most popular hypothesis for the origin of eukaryotes suggests that eukaryotes evolved by the endosymbiosis of an alphaproteobacterial ancestor of mitochondria in an archaeal host^34^, probably from the group Asgard archaea^35,36^. Since archaea do not possess calpains, while some alphaproteobacteria do, under this scenario, the host archaeal cell could have obtained calpain gene from an alphaproteobacterial endosymbiont. This scenario would be supported if alphaproteobacterial CysPC domains would be placed at the base of eukaryotic CysPCs in the phylogenetic tree with high bootstrap support. Since this is not the case (Fig. 6), our tree does not support alphaproteobacterial origin of eukaryotic calpains. Nevertheless, the hypothesis, that an archaeal ancestor of eukaryotes or the last common ancestor of eukaryotes obtained the calpain gene from an unknown bacterial donor, e.g. via an ancient horizontal gene transfer, cannot be rejected. The scenario that eukaryotic calpains are derived from genes horizontally transferred from a bacterium has been also suggested by^5^.

Rawlings (2015) has also proposed that differential distribution of calpains in bacteria is the result of multiple ancient horizontal gene transfers among bacteria rather than multiple gene losses from various bacteria^5^. In our opinion, the alternative hypothesis that both bacterial ancestor as well as eukaryotic ancestor possessed calpain can be still considered. Currently less popular but still plausible hypotheses for the origin of eukaryotes suggest that Archaea and Eukarya are sister groups. The common ancestor of Archaea and Eukarya might have originated from a bacterium^37^ or these two domains had a common undefined ancestor—a sister lineage of the domain Bacteria^38^. An undefined archaeo-eukaryotic ancestor might have been even more complex than all contemporary archaea, Archaea domain might have arisen via reductive evolution of this archaeo-eukaryotic ancestor and the differences between genome contents of contemporary archaeal lineages could be explained by differential gene losses^39–41^. Considering this scenario, the calpain gene could have been already present in the last universal common ancestor, lost in the ancestor of Archaea, while retained in the ancestor of Bacteria and in the ancestor of Eukarya. Since calpain genes are universally distributed in neither bacteria nor eukaryotes, all mentioned alternative scenarios would require multiple independent losses of calpain genes in various bacterial and eukaryotic lineages.

## Methods

We have searched for calpains in silico in proteomes of 50 selected cyanobacterial species (Supplementary Table S1). Our selection was focused on cyanobacteria from extreme biotopes as well as those with biotechnological potency. The proteomic data are available online in NCBI GenBank (https://www.ncbi.nlm.nih.gov/genbank/)^42^ and Uniprot Proteomes (https://www.uniprot.org/help/proteomes_manual) databases^43^. Since calpain superfamily is relatively divergent and it has many members, to identify potential calpain sequences, we created Hidden Markov Model (HMM) of calpain catalytic core domain (CysPC). For HMM creation, annotated calpain sequences from various organisms were obtained from the UniProt database (https://www.uniprot.org/). HMM was built using HMMER 3.2.1^44^. This model was then applied to cyanobacterial proteomes and putative calpain sequences were identified.

Putative calpains found by HMM were further analysed. Conserved domains were visualised using Conserved Domain Database (CDD) (https://www.ncbi.nlm.nih.gov/cdd)^45^ and Pfam (https://pfam.xfam.org/)^46^, and catalytic sites were identified. The sequences, which did not contain full-length CysPC domain, were excluded from analyses. The identified cyanobacterial sequences were aligned using MAFFT (https://www.ebi.ac.uk/Tools/msa/mafft/)^47^ and the sequence logo was generated using WebLogo (https://weblogo.berkeley.edu/logo.cgi)^48^. TMHMM v. 2.0 server (http://www.cbs.dtu.dk/services/TMHMM/)^49^ was used to identify putative transmembrane regions.

SmartBLAST (https://blast.ncbi.nlm.nih.gov/smartblast/) was used to search for homologs of cyanobacterial calpains in other bacteria as well as in eukaryotes. 3D structure of putative calpains was predicted by Phyre2^50^ to verify that the identified sequences are really calpains.

String DB was used for the prediction of putative interactions with other proteins^28^ to elucidate possible function of cyanobacterial calpains. String DB is the database of experimentally determined as well as predicted protein interactions. The predictions of protein interactions are based on protein homology, gene neighbourhood, gene fusions, gene co-occurrence, gene co-expression and/or text mining^28^.

The homological modelling of the 3D structure of cyanobacterial calpains was performed by Phyre2 server^50^. Supplementary Table S3 contains the list of five best-fitting models for each calpain. The model with the highest confidence and the percentual identity was selected and aligned with an appropriate template structure downloaded from the Protein Data Bank (PDB) using Multiprot server^51^. All 3D structures were visualised by ChimeraX software version 1.4 (https://www.cgl.ucsf.edu/chimerax^52^.

We gathered annotated calpain sequences from various organisms from the UniProt database (Supplementary Table S5) and we included them in phylogenetic analysis together with the identified cyanobacterial calpains (Supplementary Table S2). Only the regions corresponding to catalytic CysPC domain were used for the phylogenetic analysis. All CysPC sequences were aligned in MAFFT with automatic settings for amino acid sequences. IQ-Tree^53^ was used for the construction of phylogenetic trees. Out of 168 models, WAG + F + I + G4^54^ was selected as the best suiting model for our dataset. Bootstrap was set to 1000. Phylogenetic tree was visualized using ITOL (https://itol.embl.de/)^55^.

## Supplementary Information

Supplementary Information.

## Data Availability

All data generated or analysed during this study are included in this published article (and its Supplementary files).
