# Supplementary material for: Calpains in cyanobacteria and the origin of calpains
Source: Sci Rep. 2022 Aug 16;12:13872. doi: 10.1038/s41598-022-18228-2 (PMC9380684; doi:10.1038/s41598-022-18228-2)
Supplement: Supplementary file 1 — Supplementary Information. [file 41598_2022_18228_MOESM1_ESM.docx]

Supplementary material

***Scientific Reports***

**CALPAINS IN CYANOBACTERIA AND THE ORIGIN OF CALPAINS**

Dominika Vešelényiová^1*^, Lenka Hutárová^1^, Alexandra Lukáčová^2^, Mária Schneiderová^1^, Matej Vesteg^2^, Juraj Krajčovič^1^

^1^ Department of Biology, Faculty of Natural Sciences, University of Ss. Cyril and Methodius in Trnava, 917 01 Trnava, Slovakia

^2^ Department of Biology and Ecology, Faculty of Natural Sciences, Matej Bel University, 974 01 Banská Bystrica, Slovakia

*Correspondence: Dominika Vešelényiová, Department of Biology, Faculty of Natural Sciences, University of Ss. Cyril and Methodius in Trnava, Námestie J. Herdu 577/2, 917 01 Trnava, Slovakia, E-mail: dominika.veselenyiova@ucm.sk

https://orcid.org/0000-0002-1965-160X

Telephone: +421918804825

**Supplementary Table S1. The list of studied cyanobacteria**

| **Order** | **Genus** | **Species** | **Proteome ID** |
| --- | --- | --- | --- |
| Nostocales | *Anabaena* | *Anabaena cylindrica* | UP000010474 |
|  |  | *Anabaena minutissima* | UP000002483 |
|  | *Aphanizomenon* | *Aphanizomenon flosaquae* | UP000030828 |
|  | *Calothrix* | *Calothrix parasitica* | UP000218418 |
|  | *Raphidiopsis* | *Raphidiopsis raciborskii* | UP000052045 |
|  | *Fischerella* | *Fischerella muscicola* | UP000235036 |
|  |  | *Fischerella thermalis* | UP000004344 |
|  | *Nodularia* | *Nodularia spumigena* | UP000019325 |
|  | *Nostoc* | *Nostoc commune* | UP000245124 |
|  |  | *Nostoc cycadae* | UP000236527 |
|  |  | *Nostoc flagelliforme* | UP000232003 |
|  |  | *Nostoc linckia* | UP000217515 |
|  |  | *Nostoc minutum* | UP000252107 |
|  |  | *Nostoc punctiforme* | UP000001191 |
|  | *Scytonema* | *Scytonema hofmannii* | UP000076925 |
|  |  | *Scytonema millei* | UP000031532 |
|  |  | *Scytonema tolypothrichoide* | UP000031959 |
|  | *Trichormus* | *Trichormus azollae* | PRJNA30807 |
|  |  | *Trichormus variabilis* | UP000002533 |
| Oscillatoriales | *Arthrospira* | *Arthrospira platensis* | UP000006803 |
|  | *Coleofasciculus* | *Coleofasciculus chthonoplastes* | UP000003835 |
|  | *Microcoleus* | *Microcoleus vaginatus* | UP000004988 |
|  | *Phormidium* | *Phormidium ambiguum* | UP000185860 |
|  |  | *Phormidium willei* | UP000077169 |
| Spirulinales | *Spirulina* | *Spirulina major* | PRJNA158715 |
|  |  | *Spirulina subsalsa* | PRJNA158827 |
| Synechococcales | *Anathece* | *Anathece minutissima* | UP000236527 |
|  | *Chamaesiphon* | *Chamaesiphon minutus* | UP000010366 |
|  |  | *Chamaesiphon polymorphus* | UP000238937 |
|  | *Leptolyngbya* | *Leptolyngbya ectocarpi* | PRJNA667061 |
|  |  | *Leptolyngbya valderiana* | PRJNA312862 |
|  | *Merismopedia* | *Merismopedia glauca* | PRJNA436169 |
|  | *Phormidesmis* | *Phormidesmis priestleyi Ana* | UP000050465 |
|  | *Prochlorothrix* | *Prochlorothrix hollandica* | UP000034681 |
|  | *Stenomitos* | *Stenomitos frigidus* | UP000239576 |
|  | *Synechococcus* | *Synechococcus elongatus* | UP000002717 |
|  |  | *Synechococcus lacustris* | UP000240206 |
|  |  | *Synechococcus lividus* | UP000231057 |
|  | *Synechocystis* | *Synechocystis sp.* | UP000001425 |
|  | *Thermosynechococcus* | *Thermosynechococcus elongatus* | UP000000440 |
|  | *Vulcanococcus* | *Vulcanococcus limneticus* | PRJNA396543 |
| Chroococcidiopsidales | *Chroococcidiopsis* | *Chroococcidiopsis cubana* | UP000282574 |
|  |  | *Chroococcidiopsis thermalis* | UP000010384 |
| Chroococcales | *Aphanothece* | *Aphanothece hegewaldii* | UP000239001 |
|  |  | *Aphanothece sacrum* | UP000287247 |
|  | *Cyanosarcina* | *Cyanosarcina burmensis* | UP000238116 |
|  | *Dactylococcopsis* | *Dactylococcopsis salina* | UP000010482 |
|  | *Euhalothece* | *Euhalothece sp.* | PRJNA335957 |
|  | *Microcystis* | *Microcystis aeruginosa* | UP000001510 |
|  |  | *Microcystis flos-aquae* | PRJNA598298 |
|  |  | *Microcystis wesenbergii* | PRJNA598298 |

**Supplementary Table S2. Sequences of cyanobacterial calpains identified in this study.**

| *Anabaena minutissima* | MPIDNAGNTLNTAKKITLTTNIQTFSDWVGSSDIDDFYSLSLAGRSSLNVLVDDLNANVNAQIIKDANSNGLIDSGEIIGSYQGGYRSELIKHTLNAGNYFIRVYSKSGDTNYNLKVFENFAPTSLEFNLNSTSLHTTDNLSINNAWVSDKNGVSDISKVDFRIQRADGTWLNVADATVFTANSSDASKANFSYSLSLKNLNLAAGNYTLQGKAYDQTGAVSNTVKQTFTVTTTAPITTSTTAPTTTSTTAPTTTSTLVQDWFSQNLLDQQIITLVRSLASDGDLSRQDMLNIFRNAQDNSAIDVKEVKDLKTLLGASTPFTMQDSVKWLSTQVANGATVDMAASNFESNLVGRWFLGTIAPTPIFNGTNLTYTVPTGSLFGSSGEARIGDIDQGKLGDCTFLATLGATFGRQFNDAGNASSSLINSMITDNGDNTYTMRFYFSGAAEYVTVDRRIATSIAAKRNNGVLWVALVEKAYAQWLESRTSQPGYNVIGNGTNLFAPLQFVTGRSTTNYSISKVSFSTLETALANGQALTAARVGGDSKYIASNHAYSLTNTYIDTSGQQRVVVRNPWGVDGKTLSGSNDGFIDLSFAEFTQSFNYGISVA |
| --- | --- |
| *Aphanizomenon flosaquae* | MFDNSFDSSLSGLGNFSTYLDKDHLSSSPTVSSLQQLPSLNTPVDNAGNTLATARSVGTLTATQSFSDWVGSVDTNDYYSFNVGIQSNLTLSLTGLTANADVQLLNGSGGVITTSAKSGTTSESIASLLNTGNYFVRVYRSSGDTNYSLSLNATPIDNAGNTTATARAVGTLTATQSFSDWVGSVDTNDYYSFNVGTQSNLTLSLTGLTANADVELLNSSGTVITTAAATGTTSESITRLLSTGTYYARVYQSSGDTNYSLSLNATPVDNAGDNTGTARAVGTLTATQSFSDWVGSVDTNDYYSFNVGIQSNLTLSLTGLTANADVQLLNGSGGVITTSAKSGTTSESIASLLNTGTYFVRVYRSSGDTNYSLSLNATPIDNAGNTTATARTVGTLTATQSFSNWVGSLDTNDYYSFNVGTQSNLTLSLTGLTANADVELLNSSGTVITTAAATGTTSESITRLLSTGTYYARVYQSSGDTNYSLSLNATPVDNAGDNTGTARAVGTLTGTQSFSDWVGSVDTNDYYSFNVGIQSNLTLSLTGLTANADVQLLNGSGGVITTSAKTGTTSESIASLLNTGTYFVRVYQSSGDTNYSLSLNMIEITPNPNPTPEDWYNQNLKDAQIITLTRSLAADGNLSRNDMISIFSDAKDGSVIDANELTDLRTLVSNSTLFTMADSVKVLSNKIANSDVANTRSGFGNLSAGSNATQMENLIGKWFLGNNRPGLTSSSYSYQYVSGSLFQNGLSANDIDQGALGDCYYVATLASIAQEKPDYIQNMFTDNGDNTFTVRFYRNGVADYVTVDRYLPTNLSGRAAYAGWGGSSYTSTTNELWVALAEKAYAQLAESGWSRSSTSTNSYAAIEGGWMGSVISQVAGLGSSAADAAYMTQAQLINLVNSNQILTVGFNYAAGNTLGVVNNHAYTITAYNATNQTFHLRNPWGTRDVDVTWSQLVSLRGVMVWSNT |
| *Calothrix parasitica* | MSIENGGNAFDSPISLNTTQESQFIQGNLSSDNTNDYYSFNLTNRSSFELALNNLSDNADVKLLNENNLVVASSSRRNIQDESIRRVLNAGTYFIEVYQAGNTEIDYGLEYRSNYIPEAFQFDAEVTEGGLRLTDTKIFDADGVDDVEKVDLWLKKQGGNWNKIRNVSEFNPNDDGSIGFNYDINNLEDGKYYIWGRATDKFGARSNGWGKVFQVENFVNPEVKNVAPSNLDFDIKTVAGGIKLSDAKVYDANGVDDLERVDFQLKQEGGEWIDIQDAVDFNQNQDDSIGFDYSIANLSAGNYELKATAYDKAGNGSEALKSYFRINNIAPSDLQFEVEVIEDGIRVINTKLLDDNGISDLSRVDFWLKKDGGNWENIQDALEFRTNEDGSIGFDYSIDSLEKGNYTIWARVRDKDNKYSNSKQESFTIGNAAPTQLDFTFEQINGGIKLQDTKVFDADGTDDLEKVDFQLKKEGGEWVDIEDALNFSPNQDGSFSFEYSINGLEQGNYQLKAIASDKAGEKTKPLTTYFTVNNAAPSELLFEIETLDDGVRVVDSQVFDANGIDDLTRVDFWLKKGDNKWQNIEDAVEFRSNGNGTFSFDYSIDSLEAGDYVLWARTRDKADSYSNVWQKSFQIVDTTLESQTRQDWFSNLQDESIRELTRSRFLDNTISRSDMIAILRDAGDNNQVDETEMNDFRTIINNVSYLGIQDHVKVLSNKVVNGDVANKSGNLQVGSSTEQLNKLINKWFLGSDRPQTSHTYQYAQGSLFQNGISHDDIRQGYINDCFFLAGLGATLVQSPEIIQNMFIDNGDGTFTVRFYNKGVADYVTVDRYLPTNNIGNFVYASPGDNYADANNELWVALAEKAYAQLNESGWINQDNTNSYNGIGNAGYLSDAFAHITGERTALGRILDFEKVVNAFNSGEIVGFGSKSSGVESNIVTSHAYALVDYNSETQKFTLLNPWSTDNTALKSRTLELSWSEISSNFSYWDSTISNVVST |
| *Chamaesiphon minutus* | MFDTQNTAQNVLLGSGVQSFGGSVANLDGLDYYKLQVNNRSNVSMSLSGLGGDVNLFLLDSASRQLAASSATGIRSELIKTTLDAGTYFVKVQQATSTTSSPYQITFSNDPLFSTANSTPQSLIINGVRTSYAANSTLTLSTSYVSDSDGWQDVSKVDFWLTDRSNNRIELADVDTFTSHNAASAKFGYSTSLSQLGLAVGAYQLNAVAYDRAGVASNKFTSSAFNVINSAAQNLSISGIQSNYDSTSTLTIAPSFVSDSNGWQDVAKVDFWLTDSGNRRVELADVTSFTGNGLTSARFGYSTSLLGLASGAYKLNAVAIDKANAKSSTFTSSTFNIANSKSQDLEINGVLASYNVDDKLTLGTSYVSDNNGWRDVSKVDFWLTDRSNNRIELADVTSFSSNNLTSAKFGYSTTLTGLVVGNYNLNAVAYDKAGVASSQVMRSFSLTNAAPKTLTLNGINASYDANSTITLAPSFVSDSNGWQDVNNVDFWLTDSKGKRIELADVTSFTSNSLTTAKFDYAANLSQLGLTTGNYNLNAIAYDKSGGVSSRSVKSFAVNNTAPTTLTVNGVKSSYDLNSTLTIDPSFVTDNNGWQDVGKVDFWLTDSLNRRIELADVTSFTSDTAIAAKFGYSTSLAGLAAGNYSLNAVAYDRVGVASNTYAKSLNLVNSAPQTVTLNGLKSVYSKSSILELASSYVSDINGWQDVNKVDFWLTDSKNNRIELADVTSFTANGTNLAKFDYSTSLSALGLAAGDYNLNAVAYDKTGAASTRVSQLFNLSATLDWFDLNLKDVGVVGLARSKAADGQLDRNDLLSIFRDVQDGSVVDTSELTDLKSLMATTTPFSISDPVRYLSNKLVTDAYANINTTNFEASLGKWFLGTVAPTPTFTSSGKTTNFIYTRFQGPLFGTNTSARIGGIDQRSFGNCVLLAALGATFAPQSNDAGNSISKTINDMLLDNGDNTYTVRFFTQDLKAEWVTVDNRLATTDGKNLFGTSNKDGLWAPIIEKACAQWREFNEGSSTRTGWDIIGNGDYLDDGLQRVTGRAARNYYTGGGSWDFSFNLIKDSLSAGKAILSAGVPSVNGLNLISGHAYTVTNAYISNTGEQRVVVRNPWGIDYAWSGAADGNNDGFLDLSYDQFRTFGYITIA |
| *Chamaesiphon polymorphus* | MFDTQNTAQNVLLGSGVQSFAGSVANLDGLDYYKLQVNSRSNVSMSLSGLSDNVNLFLLDSASRQLAASSATGIRSELIKTTLEAGTYFVKVQQATSTTSSPYQLNFSNDPLFSTPNSTPQSLIVNGVKASYAANSTLTLSTSYASDRDGWQDVSKVDFWLTQSLPDSTERRIELADVDTFTSHNDASAKFGYTTSLSQLGLAVGAYKLNAVAYDKAGSTSEKFTSTAFNITNSAAQNLSISGIQTNYDATSTLTIDPSFVSDSNGWQDVSKVDFWLTNSVGRRVELADVTSFISNDGLTSARFGYSTGLLGLASGDYKLNAVAIDTANARSSTFTSSIFNIANSKPQDLQVNGVLDSYSVDSRITLATSYVSDNNGWQDVGKVDFWLTDSSNKRIELADVTSFSSNNLTSAKFGYSTALTGLAAGRYSLNALAFDKTGVTSNQFTKSFDVTNVAPKTLTLNLANTSTTPSYDANSTITLASSFVTDNNGWQDIKNVDFWLTNSKGTRIELADVTSFTSNSATTAKFDYAADLSQLGLAAGNYSLNAIAYDKSGALSSRAAKSFAVSNTAPATLTVNGVKDSYALNSTLTIDPSFVTDNNGWQDVGKVDFWLTDALNRRIELADVTSFTSDTAIAAKFGYSTSLAGLAAGSYSLNAVAYDRAGLASNTFTKSLSLVNSAPQTVTLNGLKSLYSKTSILELTSSYVTDINGWQDVTKVDFWLTDSLSRRIELADVTSFTAEGTNAKFDYSTSLSALGLAAGRYQLNAIAYDKTGAASDLAWKQFDISATLDWFDLNLKDAGVVGLARSKATDGTLDRNDMLSIFRDVQDGGVVDTSELTDLKSLMATTTPFSMSDPVRYLSNKLAIDSYANISNTAFEASLGKWFLGTVAPTATFTDESSGKVTNFTYTRFQTPLFGTNTSARIGGIDQRSFGDCVLLAALGATFAPQSNDAGNSISKTINDMLIDNGDNTYTVRFFTQDLKAEWVTVDNRLATTDGKNLFGTSNKDGLWAPIIEKACAQWREFNEGSTFYASKPATGWDIIGNGDYLDDGLQRVTGRAAKNYFTGGGSWDFSFNLIKDSLGAGKAILSAGVPSSNTLNLISGHAYTVTNAYISATGEQRVVVRNPWGIDYAWSGAADGNNDGFLDLSYTQFRNFGYITIA |
| *Fischerella thermalis 1* | MPADFAGNNLNNSRNLNINYINQTFTDWVGKGDKNDYYSFNVSSRSSLNLIVDGLSADANLQLLNSNGSVIAGSYNRKKKAETISATLDAGTYYIRVYRVNKKKSTYYNLKVSGNEAPQSLQLSTSKTSYQRGETVSLTNASVFDGNGAADLARIDFWLQKDGGEWQNIGDAVNFIANSNDNRYASFEYSLSGLSAGNYLLSAKAYDKSGASTESIQTSFSIVPVLTQDWFDLNIQDAGIREAARWHFTDHILDRNDMIAILREAKDSSVVDGTELTDLRTLVNNSSFLGMPEYVRILSHKVVNYDLANQTYQGKALGNLYAGSSDIHIENLISKWFLGSDRPTTSYNYQYAHGSLFQNGITYQDIKQGSINDCFFLTGLAATAFRSSSMIENMFIDNGDQTFTVRFYNNSIADYVTVDRYLPTNQEGYFVYASKDNYYGNSANELWVALAEKAYAQLNESGWIYQDNTNSYNGIGNGGYVSDALTNITGLNTSLANLLNFNSIVNAFNFGQMIGLTTKSTVVDANIIASHAYALIGYNSATQMFTLFNPWGVENGTSKPGIIELSWNQIEANFSYWDATINNIV |
| *Fischerella thermalis 2* | LSAGNYLLSAKAYDKSGASTESIQTSFSIVPVLTQDWFDLNIQDAGIREAARWHFTDHILDRNDMIAILREAKDSSVVDGTELTDLRTLVNNSSFLGMPEYVRILSHKVVNYDLANQNYQGKALGNLYAGSSDIHIENLISKWFLGSDRPTTSYNYQYAHGSLFQNGITYQDIKQGSINDCFFLTGLAATAFRSSSMIENMFIDNGDQTFTVRFYNNGIADYVTVDRYLPTNQEGYFVYASKDNYYGNSANELWVALAEKAYAQLNESGWIYQDNTNSYNGIGNGGYVSDALTNITGLNTSLANLLNFNSIVNAFNFGQMIGLTTKSTVVDANIIASHAYALIGYNSATQMFTLFNPWGVENGTSKPGIIELSWNQIEANFSYWDATINNIV |
| *Fischerella muscicola* | MPADFAGNNLNNSRNLNVNYINQTFTDWVGKRDKNDYYSFNVSSRSSLNLVVDSLSADANLQLLNSSGAVIAGSYNRKKKAETISTTLDAGTYYVRVYRVNKKKSTYYNLKVSGNEAPQSLQLTTSKTSYQQGETVSLTNTTIFDGNGAADLARVDFWLQKDGSEWQDIADAVNFIANSNDNRYASFEYSLSGVGAGNYLLSAKAYDKSGASTESIQTSFSIVPVPTQDWFDLNIQDAGIREAARWQFTDHILDRNDMIAILREVKDSSVVDGTELTDLRTLVNNSSFLGMPEYVRVLSNKVVNYDLANQNYQGKTLGNLYAGSSDIQIENLISKWFLGSDRPTTSYNYQYANGSLFQNGITYQDIKQGSINDCFFLTGLAATAFRSSSMIENMFIDNGDQTFTVRFYNNGIADYVTVDRYLPTNQEGYFVYASKDNYYGNSANELWVVLAEKAYAQLNESGWIYQDNTNSYNGIGNGGYVSDALANITRLNTSLANLLNYNSIVNAFNSGQMIGLTTKSTVVDANIIVAHAYALVGYNSATQMFTLFNPWGVENGTSKPGIIELSWNQIEGNFSYWDATINNIV |
| *Microcystis aeruginosa* | MLDQSFNLSDSNVLDDIFQLPTLVVESGLGRSDLITPVSGKSLQGMDSGFLSNSVEEYRLELENRWQSSQSLSSWLNGDDFLTGQSSFVGLPSSVDLAGNTLATARAITVGPSTTSYTDWVGSTDTNDYYRFTLANSGNFNLNLTGMTADADVLLLNSSGSVIASSTNGGTASESITRQLSAGTYYIRVYPYSGNTNYNLAVSATGGGNVDLAGNTLATARAITVGPSTTSYTDWVGSTDTNDYYRFTLANSGNFNLNLTGMTADADVLLLNSSGSLIASSTNGGTASESITRQLSAGTYYIRVYPYSGNTNYNLAVSAGGSDWFSQNLRDAGLISTARSLAADGNLSRNDMISIFRNAEDGSVIDATELTDLRTIVSNASRFTMQNPVVFLSNSIANGNTANQWWTGGGSTRQTLGNLYAGSSATQMERLIGKWFLGLDRPTAASGTTYRSCSGSLLQSGVSYQDINQGNLGDCYLLAALAGTAYRTPSTIQNMFTDNGDGTFTVRFFRNGVANYVTVDRYLPTTASGSFYYANRDRGLLYNNTGNELWVALAERAYAQINESGWIGQDNTNSYAGINGGWSDTTVQQITGRNTVRDYTLDNSDRTAVINAFNAGRIVFLNWAGHALTLVGYNPTTQLFTIYNPWGHADNFTWNQIINGTGTGHEQRFTDWSYTTT |
| *Scytonema hofmannii 1* | MQNDFTSHALHTSLSINITASHQTFAGSLDRRNPNDYYSFSLNGSSSLSLSLDGLSADADVQLLDSNGSTITGSHNRHNTAESLGITVGAGTYYIEVSRVGSANTSYNLKAFKNEAPQSLQFNTYKGSYEAGETVNLTNTRVFDANGANDLARVDFWLQKDGSNWQDIHDVLEFSVDNTDSCYSSFTYCLNSLSSGNYQLLAKAYDKLGASTQSTQTSFKVGAALDWFDQNIQDEVIRSAARSRFSDGLLDRNDMVSILRESKDGNVVDGTELADLRTLISNTSYINISEHVRVLSNKVLNNDTANQKYQGNSLGNLVAGSSDVQLENLINKWFFGSDRPQSSYTYQYASGSLFQNGINYEDVKQGSMNDCYFLAGLASTASRTPNTIESMFIDNGDNTFTVRFWRSGVADYVTVDKYLPTDASGAFIYASKGSHYSNASNELWVAFAEKAYAQLNESGWIFQNNTNTYEGIGKGGYMSDAFAQITGKKAALGKAIDCNSIINAFNSGQLVGLGTKTTGVGANIVAGHAYSLVGYNSSTQKFALFNPWGINTSSSKPGVIEVSWSEVQSNFSYWDTTV |
| *Scytonema hofmannii 2* | MTRQRSNSAPPRLPTQETAVLQSEAGSSNWRQDATNSQQTTTSLRNLDLRSINSYSHPLSSHEQNDDFDEILDLYDQRETDEDATNSQQTTTNSRNLDLSPINSDSHPPLSHEQNDDFDEILNIHDQRETDEEQNDENNEERYKTNENALEKPLRKPDRLRLNQAQVTTTEGKEETISKEQNFKITDTEEDETSYIIQYYKWNSNNSEPRQRLEEIQGRIEKDNVVKGSRQQTPLENINTGRRNRPVQILPNNVRGIRQEDVYQNGVSDCYLQAAMIAVAKQNPQQIWDMVSVNDNDVRVTFHLPENNENNASNLNQELRPIEITVKKSLLLNNDGQLVYGGKIGNNNYLWPAFIQKAWAVVKGGYAESAMGVVTDTIKAITGEGRSTPFRLTHHWNIYNNLVTALNDRRAAVLTTSVFAKQGKLKKLLNSFNDRSAPKDKPLGKVRVMHAYAVLDMDNRNLTENDFQDNAIPDVSLTLRDPRDPKGKTFKRTLKQVLSKDKFDAVHTG |
| *Scytonema hofmannii 3* | MLNSFSIDEYAPLIEGFDPYVLVNRGEDITGSNFNSVEAGYAQTNWDTYLSPQPVDWFGLTGYSAQPSHEFGNGNSSTQTTDWFSQNLIDPQLITKARTLAADSQFNRNDMMAIFRDAKDGNVIDANELKDLRTLVSNANRFNIPDDVRVLSNKIVNGNAANIKYQGQNLGNLFAGSTGDQMEKLIGKWFLGSDRPTIPTNYTYRQANGYLFQNGVSYQDVKQGGLADCYFMAGLAEVALHSPSKIQSMFTDNGDNTYTVRFYNSGVADYVTVDKYLPTNSSGNFVYAQQAKSDGGNYKDLSNELWVALAEKAYAQVNESGWIGQDNTNSYLGIEYGADNKVITHVTGRNASSSSLDFNSMVNAYKAGNLMGVSSYNSGVASNIAPNHVYVVTNYDSFTQQFTLYNPWGLDGGNYEGKFKPGTIKLSFNELKASFASWSYTT |
| *Trichormus variabilis* | MTLDNAGNTLTTARKLTVSSNIQTFADRVDSTDPNDFYSFSLSARSSLNIAVDGLSANADLQLIRDTNSNGLVDSGEVLNTSNKTGTGSESIRRTLDAGKYFIRVYSNTGDTNYNLKVFENFTPTSLEFKLNESTLKATDTLNINSGWVSDRNGISDLSKVDFRIQRANGSWIDVADATQFTVDPNNTNKAGFSYSLSLNSLNLAADTYTLQGIAYDKTGAASNTVRLSLTIENPGLTLTNAKKITLSEKTQTFTDRVDSTNINDFYSFSLSARGNLNLVVDGLSASADVQIIRDANSNGLFDGGEVVTGAYRTGSGSESIRTTLDAGNYFIRVYSQGGNTNYKLKVFENFAPTALDFKLNNTSLKPTDTLSINSAWVSDKNGVSDISKVDFRIQKADGSWIDVADATKFTADSSNANKASFSYSLSLSSLNLAVGTYTLQGIAYDKTNAASNTVKQTFTVTTTPTTTASATVQDWFSQNLLDQQLITLTRNLAADGNLSRQDMLDIFRNVQDDSKVDANEVKDLRTLVGASTRFSMQDPVKWLSTQVANGASVDMAASDFESSLVGRWFLGTVAPTPVFNGKTLTYTLATGNLFGSANEARIGDIDQGQLGDCAFLAALGATFGRQSNDAGNASSSVINSMITDNGDNTYTVRFYSTTIFDPGEAQYVTIDRRIATSVAAKTNGGVLWVALVEKAYAQWREWREGKPGYNIIGNGDALSRPLQFVTGRDFTPADPTNINCFSTIETALANGKAVTAARMGDSTSYIVGNHAYSVTNVYTNTSGEKRFVVRNPWGKDGKTRTGADDGFIDLSFDEFSKAFNYGVIIA |

**Supplementary Table S3. 3D structure modelling of cyanobacterial CysPC domains and the list of top five templates for each cyanobacterial calpain.** Templates selected for further analyses are highlighted in grey.

| **Cyanobacterium** | **Conserved domain** |  | **Template ID** | **Confidence** | **%i.d.** | **Template Information** |
| --- | --- | --- | --- | --- | --- | --- |
| *Anabaena minutissima* | CysPC | 1 | d1kful3 | 100 | 28 | Calpain large subunit, catalytic domain (domain II) |
| *Anabaena minutissima* | CysPC | 2 | d1mdwa | 100 | 28 | Calpain large subunit, catalytic domain (domain II) |
| *Anabaena minutissima* | CysPC | 3 | d1ziva1 | 100 | 27 | Calpain large subunit, catalytic domain (domain II) |
| *Anabaena minutissima* | CysPC | 4 | c1zivA | 100 | 27 | Catalytic domain of human calpain-9 |
| *Anabaena minutissima* | CysPC | 5 | c1kfxL | 100 | 28 | Crystal structure of human m-calpain form i |
| *Calothrix parasitica* | CysPC | 1 | c6p3qB | 100 | 26 | Calpain-5 (capn5) protease core (pc) |
| *Calothrix parasitica* | CysPC | 2 | d2r9fa1 | 100 | 25 | Calpain large subunit, catalytic domain (domain II |
| *Calothrix parasitica* | CysPC | 3 | d1kful3 | 100 | 25 | Calpain large subunit, catalytic domain (domain II) |
| *Calothrix parasitica* | CysPC | 4 | c6bdtC | 100 | 25 | Crystal structure of human calpain-3 protease core mutant |
| *Calothrix parasitica* | CysPC | 5 | d1mdwa | 100 | 25 | Calpain large subunit, catalytic domain (domain II) |
| *Chamaesiphon minutus* | CysPC | 1 | c1kfxL | 100 | 30 | Crystal structure of human m-calpain form i |
| *Chamaesiphon minutus* | CysPC | 2 | d1mdwa | 100 | 28 | Calpain large subunit, catalytic domain (domain II) |
| *Chamaesiphon minutus* | CysPC | 3 | d2r9fa1 | 100 | 27 | Calpain large subunit, catalytic domain (domain II) |
| *Chamaesiphon minutus* | CysPC | 4 | d1zcma1 | 100 | 25 | Calpain large subunit, catalytic domain (domain II) |
| *Chamaesiphon minutus* | CysPC | 5 | c1qxpB | 100 | 25 | Crystal structure of a mu-like calpain |
| *Fischerella muscicola* | CysPC | 1 | d1kful3 | 100 | 27 | Calpain large subunit, catalytic domain (domain II) |
| *Fischerella muscicola* | CysPC | 2 | d2r9fa1 | 100 | 27 | Calpain large subunit, catalytic domain (domain II) |
| *Fischerella muscicola* | CysPC | 3 | c6p3qB | 100 | 26 | Calpain-5 (capn5) protease core (pc) |
| *Fischerella muscicola* | CysPC | 4 | c6bdtC | 100 | 26 | Crystal structure of human calpain-3 protease core mutant |
| *Fischerella muscicola* | CysPC | 5 | c1qxpB | 100 | 26 | Crystal structure of a mu-like calpain |
| *Fischerella thermalis 2* | CysPC | 1 | c6p3qB | 100 | 27 | Crystal structure of a mu-like calpain |
| *Fischerella thermalis 2* | CysPC | 2 | c1qxpB | 100 | 26 | Calpain-5 (capn5) protease core (pc) |
| *Fischerella thermalis 2* | CysPC | 3 | c6bdtC | 100 | 26 | Crystal structure of human calpain-3 protease core mutant |
| *Fischerella thermalis 2* | CysPC | 4 | d1kful3 | 100 | 25 | Calpain large subunit, catalytic domain (domain II) |
| *Fischerella thermalis 2* | CysPC | 5 | d2r9fa1 | 100 | 25 | Calpain large subunit, catalytic domain (domain II) |
| *Scytonema hofmannii 1* | CysPC | 1 | c6bdtC | 100 | 29 | Crystal structure of human calpain-3 protease core mutant-c129s |
| *Scytonema hofmannii 1* | CysPC | 2 | d1kful3 | 100 | 28 | Calpain large subunit, catalytic domain (domain II) |
| *Scytonema hofmannii 1* | CysPC | 3 | c6p3qB | 100 | 26 | Calpain-5 (capn5) protease core (pc) |
| *Scytonema hofmannii 1* | CysPC | 4 | c1kfxL | 100 | 28 | Crystal structure of human m-calpain form i |
| *Scytonema hofmannii 1* | CysPC | 5 | d1mdwa | 100 | 27 | Calpain large subunit, catalytic domain (domain II) |
| *Scytonema hofmannii 3* | CysPC | 1 | d1qxpa4 | 100 | 30 | Calpain large subunit, catalytic domain (domain II) |
| *Scytonema hofmannii 3* | CysPC | 2 | c6bdtC | 100 | 29 | Crystal structure of human calpain-3 protease core mutant |
| *Scytonema hofmannii 3* | CysPC | 3 | d1kful3 | 100 | 29 | Calpain large subunit, catalytic domain (domain II) |
| *Scytonema hofmannii 3* | CysPC | 4 | d2r9fa1 | 100 | 29 | Calpain large subunit, catalytic domain (domain II) |
| *Scytonema hofmannii 3* | CysPC | 5 | d1zcma1 | 100 | 29 | Calpain large subunit, catalytic domain (domain II) |
| *Trichormus variabilis* | CysPC | 1 | c1kfxL | 100 | 32 | Crystal structure of human m-calpain form i |
| *Trichormus variabilis* | CysPC | 2 | d1mdwa | 100 | 30 | Calpain large subunit, catalytic domain (domain II) |
| *Trichormus variabilis* | CysPC | 3 | d1kful3 | 100 | 29 | Calpain large subunit, catalytic domain (domain II) |
| *Trichormus variabilis* | CysPC | 4 | c6p3qB | 100 | 27 | Calpain-5 (capn5) protease core (pc) |
| *Trichormus variabilis* | CysPC | 5 | d2r9fa1 | 100 | 26 | Calpain large subunit, catalytic domain (domain II) |
| *Fischerella thermalis 1* | CysPC | 1 | d1mdwa | 100 | 29 | Calpain large subunit, catalytic domain (domain II) |
| *Fischerella thermalis 1* | CysPC | 2 | c6bdtC | 100 | 27 | Crystal structure of human calpain-3 protease core mutant-c129 |
| *Fischerella thermalis 1* | CysPC | 3 | c1qxpB | 100 | 27 | Crystal structure of a mu-like calpain |
| *Fischerella thermalis 1* | CysPC | 4 | d1kful3 | 100 | 26 | Calpain large subunit, catalytic domain (domain II) |
| *Fischerella thermalis 1* | CysPC | 5 | d2r9fa1 | 100 | 26 | Calpain large subunit, catalytic domain (domain II) |
| *Microcystis aeruginosa* | CysPC | 1 | d1kful3 | 100 | 32 | Calpain large subunit, catalytic domain (domain II) |
| *Microcystis aeruginosa* | CysPC | 2 | d2r9fa1 | 100 | 32 | Calpain large subunit, catalytic domain (domain II) |
| *Microcystis aeruginosa* | CysPC | 3 | d1zcma1 | 100 | 32 | Calpain large subunit, catalytic domain (domain II) |
| *Microcystis aeruginosa* | CysPC | 4 | c1qxpB | 100 | 31 | Crystal structure of a mu-like calpain |
| *Microcystis aeruginosa* | CysPC | 5 | c6bdtC | 100 | 31 | Crystal structure of human calpain-3 protease core mutant-c129s |
| *Aphanizomenon flosaquae* | CysPC | 1 | c6p3qB | 100 | 32 | Calpain-5 (capn5) protease core (pc) |
| *Aphanizomenon flosaquae* | CysPC | 2 | d1qxpa4 | 100 | 32 | Calpain large subunit, catalytic domain (domain II) |
| *Aphanizomenon flosaquae* | CysPC | 3 | d2r9fa1 | 100 | 30 | Calpain large subunit, catalytic domain (domain II) |
| *Aphanizomenon flosaquae* | CysPC | 4 | c6bdtC | 100 | 30 | Crystal structure of human calpain-3 protease core mutant-c129s |
| *Aphanizomenon flosaquae* | CysPC | 5 | d1zcma1 | 100 | 30 | Calpain large subunit, catalytic domain (domain II) |
| *Chamaesiphon polymorphus* | CysPC | 1 | c1kfxL | 100 | 31 | Crystal structure of human m-calpain form i |
| *Chamaesiphon polymorphus* | CysPC | 2 | d1kful3 | 100 | 30 | Calpain large subunit, catalytic domain (domain II) |
| *Chamaesiphon polymorphus* | CysPC | 3 | d1mdwa | 100 | 29 | Calpain large subunit, catalytic domain (domain II) |
| *Chamaesiphon polymorphus* | CysPC | 4 | d1zcma1 | 100 | 26 | Calpain large subunit, catalytic domain (domain II) |
| *Chamaesiphon polymorphus* | CysPC | 5 | c1zivA | 100 | 26 | Catalytic domain of human calpain-9 |
| *Scytonema hofmannii 2* | CysPC | 1 | d1zcma1 | 100 | 24 | Calpain large subunit, catalytic domain (domain II) |
| *Scytonema hofmannii 2* | CysPC | 2 | d2r9fa1 | 100 | 22 | Calpain large subunit, catalytic domain (domain II) |
| *Scytonema hofmannii 2* | CysPC | 3 | c1qxpB | 100 | 24 | Crystal structure of a mu-like calpain |
| *Scytonema hofmannii 2* | CysPC | 4 | c1zivA | 100 | 23 | Catalytic domain of human calpain-9 |
| *Scytonema hofmannii 2* | CysPC | 5 | d1ziva1 | 100 | 23 | Calpain large subunit, catalytic domain (domain II) |

**Supplementary Table S4. Information about 3D structure comparison of cyanobacterial CysPC domains and Protein Data Bank template.** Multiprot alignment – number of aligned amino acids, Multiprot RMSD – Root mean quare deviation, %i.d. – percentual identity

| **Cyanobacterium** | **Conserved domain** | **Template ID** | **Confidence** | **%i.d.** | **Template Information** | **Multiprot alignment** | **Miltiprot RMSD** |
| --- | --- | --- | --- | --- | --- | --- | --- |
| *Anabaena minutissima* | CysPC | d1kful3 | 100 | 28 | Calpain large subunit, catalytic domain (domain II) | 189 | 0.82 |
| *Aphanizomenon flosaquae* | CysPC | c6p3qB | 100 | 32 | Calpain-5 (capn5) protease core (pc) | 182 | 0.63 |
| *Calothrix parasitica* | CysPC | c6p3qB | 100 | 26 | Calpain-5 (capn5) protease core (pc) | 227 | 0 |
| *Chamaesiphon minutus* | CysPC | c1kfxL | 100 | 30 | Crystal structure of human m-calpain form i | 197 | 0.77 |
| *Chamaesiphon polymorphus* | CysPC | c1kfxL | 100 | 31 | Crystal structure of human m-calpain form i | 184 | 0.71 |
| *Fischerella muscicola* | CysPC | d1kful3 | 100 | 27 | Calpain large subunit, catalytic domain (domain II) | 207 | 0.59 |
| *Fischerella thermalis* 1 | CysPC | d1mdwa | 100 | 29 | Calpain large subunit, catalytic domain (domain II) | 177 | 0.62 |
| *Fischerella thermalis* 2 | CysPC | c6p3qB | 100 | 27 | Crystal structure of a mu-like calpain | 203 | 0.7 |
| *Microcystis aeruginosa* | CysPC | d1kful3 | 100 | 32 | Calpain large subunit, catalytic domain (domain II) | 180 | 0.76 |
| *Scytonema hofmannii* 1 | CysPC | c6bdtC | 100 | 29 | Crystal structure of human calpain-3 protease core mutant-c129s | 220 | 0.7 |
| *Scytonema hofmannii* 2 | CysPC | d1zcma1 | 100 | 24 | Calpain large subunit, catalytic domain (domain II) | 216 | 0.65 |
| *Scytonema hofmannii* 3 | CysPC | d1qxpa4 | 100 | 30 | Calpain large subunit, catalytic domain (domain II) | 182 | 0.64 |
| *Trichormus variabilis* | CysPC | c1kfxL | 100 | 32 | Crystal structure of human m-calpain form i | 180 | 0.66 |

**Supplementary Table S5. Sequences used for phylogenetic analysis.** Sequences of calpains identified in this study are listed in Supplementary Table S2. Bacteria belonging to neither alphaproteobacteria, nor cyanobacteria are assigned as “other bacteria”.

| **Organism** | **Taxon** | **UniProt Entry** |
| --- | --- | --- |
| Sequences available in UniProt | | |
| *Acetobacter cibinongensis* | alphaproteobacteria | A0A1Z5YT36 |
| *Allonocardiopsis opalescens* | other bacteria | A0A2T0Q9Z4 |
| *Ancylobacter aquaticus* | alphaproteobacteria | A0A431QS00 |
| *Ancylobacter rudongensis* | alphaproteobacteria | A0A1G4PC52 |
| *Arabidopsis thaliana* | Streptophyta | Q8RVL2 |
| *Aspergillus oryzae* | Fungi | Q2UM54 |
| *Bacteroides thetaiotaomicron* | other bacteria | R9H254 |
| *Brachybacterium faecium* | other bacteria | C7MGJ8 |
| *Bradyrhizobium uaiense* | alphaproteobacteria | A0A6P1BHT8 |
| *Chlamydomonas reinhardtii* | Chlorophyta | A0A2K3E334 |
| *Chrysochromulina tobinii* | Haptophyta | A0A0M0JT31 |
| *Danio rerio* | Metazoa | B8A6G0 |
| *Drosophila melanogaster* | Metazoa | Q11002 |
| *Ectocarpus siliculosus* | SAR | D7FTW8 |
| *Emiliania huxleyi* | Haptophyta | R1F5H3 |
| *Entamoeba histolytica* | Amoebozoa | C4LTE2 |
| *Frankia alni* | other bacteria | Q0RAZ2 |
| *Gemmata obscuriglobus* | other bacteria | A0A2Z3H2V8 |
| *Geobacter lovleyi* | other bacteria | B3E5A8 |
| *Haloactinopolyspora alba* | other bacteria | A0A2P8DJ78 |
| *Homo sapiens* | Metazoa | P07384 |
| *Lichenicoccus roseus* | alphaproteobacteria | A0A5R9JC88 |
| *Magnetococcus_massalia* | other bacteria | A0A1S7LJK3 |
| *Murinocardiopsis flavida* | other bacteria | A0A2P8DFD3 |
| *Neurospora crassa* | Fungi | Q7RZW0 |
| *Physcomitrella patens* | Streptophyta | A0A2K1J4D2 |
| *Planoprotostelium fungivorum* | Amoebozoa | A0A2P6NJ62 |
| *Pseudoxanthobacter soli* | alphaproteobacteria | A0A1M7ZL25 |
| *Rhodobacteraceae bacterium* | alphaproteobacteria | A0A2D7JI98 |
| *Rhodoblastus acidophilus* | alphaproteobacteria | A0A212S3E6 |
| *Rhodoblastus sphagnicola* | alphaproteobacteria | A0A2S6N9W1 |
| *Rhodopila globiformis* | alphaproteobacteria | A0A2S6NJ85 |
| *Rhodopseudomonas palustris* | alphaproteobacteria | Q07SP7 |
| *Rhodovastum atsumiense* | alphaproteobacteria | A0A5M6IQ67 |
| *Segniliparus rotundus* | other bacteria | D6ZC85 |
| *Siculibacillus lacustris* | alphaproteobacteria | A0A4Q9VDM5 |
| *Sphingopyxis macrogoltabida* | alphaproteobacteria | A0A0P0DHN5 |
| *Streblomastix strix* | Metamonada | A0A5J4UD17 |
| *Streptomyces viridochromogenes* | other bacteria | D9X719 |
| *Tetrahymena thermophila* | SAR | Q24DF0 |
| *Thecamonas trahens* | Apusozoa | A0A0L0DIP0 |
| *Tritrichomonas foetus* | Metamonada | A0A1J4JR60 |
| *Vitrella brassicaformis* | SAR | A0A0G4FDZ9 |
| *Volvox carteri* | Chlorophyta | D8UEL0 |
| *Zea mays* | Streptophyta | Q8RVL1 |
| Sequences indentified in this study: | | |
| *Anabaena minutissima* | cyanobacteria |  |
| *Aphanizomenon flosaquae* | cyanobacteria |  |
| *Calothrix parasitica* | cyanobacteria |  |
| *Chamaesiphon minutus* | cyanobacteria |  |
| *Chamaesiphon polymorphus* | cyanobacteria |  |
| *Fischerella muscicola* | cyanobacteria |  |
| *Fischerella thermalis 1* | cyanobacteria |  |
| *Fischerella thermalis 2* | cyanobacteria |  |
| *Microcystis aeruginosa* | cyanobacteria |  |
| *Scytonema hofmannii 1* | cyanobacteria |  |
| *Scytonema hofmannii 2* | cyanobacteria |  |
| *Scytonema hofmannii 3* | cyanobacteria |  |
| *Trichormus variabilis* | cyanobacteria |  |





**Supplementary Fig. S1.** **Prediction of interaction partners for cyanobacterial calpains by String DB.** Some predicted interaction partners are not annotated (NA). Cyanobacterial species and identified calpains are shown in red and annotated proteins in green frames. The type of evidence for each interaction is indicated by the colour of the connecting lines: experimentally determined (pink), information from curated databases (light blue), gene neighbourhood (green), gene fusions (red), gene co-occurrence (dark blue), text mining (yellow), co-expression (black) and protein homology (purple).
